# PSYCHOLOGICAL IMPACT OF COVID-19 LOCKDOWN ON A POPULATION WITH SERIOUS MENTAL DISORDER: DIAGNOSTIC GROUP ANALYSIS

**DOI:** 10.1192/j.eurpsy.2023.1644

**Published:** 2023-07-19

**Authors:** B. Pedruzo, C. Aymerich, A. Catalan, M. Pacho, M. Bordenave, O. Estevez, J. Herrero, M. Laborda, G. Mancebo, J. L. Perez, M. A. Gonzalez Torres

**Affiliations:** 1 Service of Psychiatry. Basurto University Hospital, Osakidetza, Bilbao, Spain

## Abstract

**Introduction:**

Since its emergence at the end of 2019, the COVID-19 virus has spread worldwide. In Spain, mandatory home confinement was established on March 15, 2020, and lasted 99 days. Previous studies on events that required isolation situations suggest a worsening in the mental health of general population, and in particular, of especially vulnerable groups such as individuals with severe mental disorder (SMD).

**Objectives:**

The aim of this study is to evaluate the psychological effect (anxiety and depression) of confinement in patients with SMD and to study the dissimilarities among the different diagnostic groups.

**Methods:**

In this study, assessments were performed using the IDER and STAI questionnaires, in order to evaluate symptoms of depression and anxiety, respectively. The evaluations were carried out in patients who had required at least one admission to the Psychiatric Hospitalization Unit of the University Hospital of Basurto. The Shapiro-Wilk test was used to verify the normality of the sample. ANOVA test was used to study differences among diagnostic groups. Posteriorly, Bonferroni correction was performed.

**Results:**

95 participants completed the IDER questionnaire, obtaining a mean score of 24.56 (SD=8.18) for the state and 23.57 (SD=8.14) for the trait. In the STAI questionnaire, a mean score of 27.86 (SD=15.19) was obtained for the state and 30.49 (SD=14.71) for the trait. ANOVA test indicated presence of differences among groups. However, differences did not persist after Bonferroni correction.

**Conclusions:**

Increased levels of anxiety and depression were found in the sample studied with respect to the general population.No statistically significant differences were found among different disgnostic groups. Further studies should be performed in order to increase the knowledge around this research area.

**Disclosure of Interest:**

None Declared

